# Unraveling the Role of Bromodomain and Extra-Terminal Proteins in Human Uterine Leiomyosarcoma

**DOI:** 10.3390/cells13171443

**Published:** 2024-08-28

**Authors:** Qiwei Yang, Ali Falahati, Azad Khosh, Ricardo R. Lastra, Thomas G. Boyer, Ayman Al-Hendy

**Affiliations:** 1 Department of Obstetrics and Gynecology, University of Chicago, Chicago, IL 60637, USA; aalhendy@bsd.uchicago.edu; 2 Poundbury Cancer Institute for Personalised Medicine, Dorchester DT1 3BJ, UK; ali.falahati@nhs.net; 3 DNA GTx LAB, Dubai Healthcare City, Dubai 505262, United Arab Emirates; 4 Department of Molecular Medicine, Institute of Biotechnology, University of Texas Health Science Center at San Antonio, San Antonio, TX 78229, USA; khosh@livemail.uthscsa.edu (A.K.); boyer@uthscsa.edu (T.G.B.); 5 Department of Pathology, University of Chicago, Chicago, IL 60637, USA; ricardo.lastra@uchospitals.edu

**Keywords:** uterine leiomyosarcoma, bromodomain and extra-terminal protein, transcriptome analysis, epigenome, m^6^A regulators, JQ1, I-BET 762, hedgehog pathway, EMT, transcriptional factors

## Abstract

Uterine leiomyosarcoma (uLMS) is the most common type of uterine sarcoma, associated with poor prognosis, high rates of recurrence, and metastasis. Currently, the molecular mechanism of the origin and development of uLMS is limited. Bromodomain and extra-terminal (BET) proteins are involved in both physiological and pathological events. However, the role of BET proteins in the pathogenesis of uLMS is unknown. Here, we show for the first time that BET protein family members, BRD2, BRD3, and BRD4, are aberrantly overexpressed in uLMS tissues compared to the myometrium, with a significant change by histochemical scoring assessment. Furthermore, inhibiting BET proteins with their small, potent inhibitors (JQ1 and I-BET 762) significantly inhibited the uLMS proliferation dose-dependently via cell cycle arrest. Notably, RNA-sequencing analysis revealed that the inhibition of BET proteins with JQ1 and I-BET 762 altered several critical pathways, including the hedgehog pathway, EMT, and transcription factor-driven pathways in uLMS. In addition, the targeted inhibition of BET proteins altered several other epigenetic regulators, including DNA methylases, histone modification, and m^6^A regulators. The connections between BET proteins and crucial biological pathways provide a fundamental structure to better understand uterine diseases, particularly uLMS pathogenesis. Accordingly, targeting the vulnerable epigenome may provide an additional regulatory mechanism for uterine cancer treatment.

## 1. Introduction

Uterine leiomyosarcoma (uLMS) is the most common type of uterine sarcoma arising from smooth muscle cells [1,2]. The 5-year survival rate for all uLMS patients ranges between 25 and 76%, while survival for women at an advanced stage of the disease at the time of initial diagnosis approaches only 10–15% [3]. uLMS is a heterogeneous aggressive disease with a poor prognosis and a challenging clinical treatment outlook [4]. Accordingly, uLMS patients present resistance to available chemotherapies, as evidenced by high rates of both recurrence and progression [5,6]. There is an urgent need to develop effective strategies to improve the outcome for patients with uLMS.

Cumulative data strongly demonstrate that the epigenetic dysregulation of gene expression contributes to the development of various tumors [7,8,9,10,11,12,13]. DNA methylation profiling studies showed that uLMS displays a distinct methylation signature [14]. In addition, non-coding RNAs have been investigated and their crucial role in regulating multiple cellular signaling processes in uLMS has been demonstrated [15,16]. Histone modifications, as one of the key epigenetic regulatory features, are dynamically modulated by “writers” and “erasers”. In addition, distinct effector proteins (“readers”) can recognize specific histone modifications and regulate gene expression. Bromodomain-containing proteins (BRDs), as epigenetic reader proteins, are responsible for transducing regulatory signals carried by acetylated lysine residues into various biological phenotypes [17]. BRDs can act as scaffolds that enable the recruitment of large protein complexes, or they can act as transcription factors themselves. BRDs contain several catalytic domains that enable them to act as methyltransferases, ATP-dependent chromatin re-modelers, or histone acetyltransferases and helicases [18,19]. Notably, BRD9 was recently reported to contribute to the uLMS pathogenesis [5].

So far, over sixty BRDs have been identified in the human genome, and these BRDs were divided into eight subfamilies (e.g., bromodomain and extra-terminal domain [BET] subfamilies) based on protein sequence similarity [20,21]. The BET family of proteins, characterized by the presence of two tandem bromodomains and an extra-terminal domain, consists of BRD2, BRD3, BRD4, and BRDT, which bind histone acetylated lysine residues via two highly conserved amino-terminal bromodomains [19]. BRDs are specifically capable of binding acetylated lysine residues in histones, serving as chromatin-targeting modules that decipher the histone acetylation code. BET proteins regulate gene transcription through epigenetic interactions between bromodomains and acetylated histones during cellular proliferation and differentiation [22]. Studies have demonstrated that BET proteins play an oncogenic role in multiple cancer types by regulating tumor cell growth and metastasis. The connection of BET proteins with key oncogenic transcription factor pathways, chronic inflammation, and immune surveillance is implicated in cancer progression.

Gynecologic cancers include cervical cancer, ovarian cancer, vulvar cancer, vaginal cancer, fallopian tube cancer, and uterine cancer. Growing evidence demonstrates that BET BRDs play an oncogenic role in gynecological cancer, including ovarian cancer. For example, BRD4, located at chromosome 19p13, was amplified in a considerable proportion (~20%) of ovarian cancers, and the expression of BRD4 correlated with amplification status [23]. Recent reports demonstrated that BRD4 is directly involved in the metastatic process in high-grade serous ovarian carcinoma [24]. The pharmacological inhibition of BRD4 with BET inhibitors (BETis) JQ1 and I-BET151 substantially abrogated both the in vitro growth and in vivo tumorigenesis of ovarian cancer [23], and BRD4 inhibitors have been largely used in several pre-clinical studies in ovarian cancer [25,26,27]. Notably, combination therapy with BET inhibitors effectively overcome chemoresistance in pre-clinical settings [25,28]. Concomitant BRD4 inhibition with PARP inhibitor [29], cisplatin [30], tyrosine kinase inhibitors [31,32], and MEK inhibitors [26,33] has been shown to efficiently and synergistically suppress ovarian carcinoma growth. These studies emphasize the critical impact of abnormal BET protein function on human diseases, especially in gynecological cancer. Therefore, the targeted inhibition of BET proteins may provide a promising option for treating patients with gynecological cancers [20]. However, current knowledge regarding the role and mechanism of BET proteins in the pathogenesis of uterine cancer, such as uLMS, is limited. Accordingly, the present study aimed to investigate whether and how BET proteins contribute to aberrant uLMS cell growth, with important implications for developing novel treatment options for this highly aggressive uterine cancer.

## 2. Materials and Methods

### 2.1. Uterine Leiomyosarcoma Samples

The experimental design and flowchart of the bioinformatics analysis are shown in Figure S1. The uLMS tissues were obtained from the University of Chicago Tissue Bank with the approval and consent of the Institutional Review Board (# 20-1820) at the University of Chicago. The initial diagnosis and subsequent confirmation of uLMS cases were described previously [5].

### 2.2. Immunohistochemistry

Immunohistochemistry was performed as previously described [5]. Briefly, sections were deparaffinized with xylene and rehydrated passing through decreasing concentrations of ethanol in water. Then, antigen retrieval treatment (epitope retrieval solution II, AR9640, Leica Biosystems, Dear Park, IL, USA) was performed for 20 min, and the quenching of endogenous peroxidases was performed. Next, sections were incubated with primary antibodies against BRD2 (Abcam, ab139690), BRD3 (Abcam, Ab264420), and BRD4 (Abcam, Ab128874) in a humidity chamber overnight at 4 °C and developed with peroxidase labeled-dextran polymer followed by diaminobenzidine (DAKO Envision Plus System; DAKO Corporation, Carpinteria, CA, USA). Sections were counterstained with Gill’s Hematoxylin (Fisher, Pittsburgh, PA, USA). To determine the percentage and intensity of BET protein-positive cells, the samples were analyzed using the positive cell detection command on QuPath software (version 0.2.3) (https://qupath.github.io, accessed on 28 November 2022). Three thresholds were set to categorize cells according to nuclei staining intensity: negative, weak, moderate, and strong intensity. The H-score captures the intensity and the proportion of the biomarker of interest from the IHC image and comprises values between 0 and 300 [34], thereby offering a dynamic range to quantify BET protein abundance between myometrium and uLMS. Human testis was used as a positive control for BRD2, BRD3, and BRD4 IHC staining.

### 2.3. Cells and Reagents

The uterine leiomyosarcoma (uLMS) cell line (SK-UT-1, ATCC^®^ HTB-114^TM^) (ATCC, Manassas, VA, USA) was cultured and maintained in ATCC-formulated Eagle’s Minimum Essential Medium with 10% of fetal bovine serum. The immortalized human uterine smooth muscle (UTSM) cell line was a generous gift from Dr. Darlene Dixon. The UTSM cells were cultured and maintained in phenol red-free, 10% fetal bovine serum Dulbecco’s Modified Eagle Medium: Nutrient Mixture F-12. BET protein inhibitors JQ1 and I-BET 762 were purchased from Tocris (Cat# 4499 and 6521, Minneapolis, MN, USA).

### 2.4. Cell Viability Assay

Cell viability was measured using trypan blue exclusion assay. The cells were seeded into 12-well tissue culture plates and treated with the BET protein inhibitors (JQ1, I-BET 762) at a dose range from 1–10 µM for 48 h. This assay was performed three times in triplicate.

### 2.5. Measurement of Cell Cycle Phase Distribution

Cell cycle distribution was determined by flow cytometric analysis as described previously [35]. Briefly, SK-UT-1 cells were cultured in the medium in the presence or absence of 5 µM of JQ1 and I-BET 762 for 24 h. Control cells were cultured in a medium containing an equal amount of DMSO. Cells were fixed with 70% ethanol and then hypotonically lysed in DNA staining solution (0.05 mg/mL PI (Sigma, St. Louis, MO, USA) and 0.1% Triton X-100). The flow cytometry analysis was performed to determine the difference in cell cycle distribution between treated and untreated groups.

### 2.6. RNA Extraction and Gene Expression

Total RNA extraction and quantitative real-time polymerase chain reaction (qRT-PCR) were performed as described previously [11]. The information about primer sequences is shown in Table S1.

### 2.7. RNA-Sequencing

We performed whole transcriptome analysis to determine the mechanism underlying the inhibitory effect of BET protein inhibition on the uLMS. The SK-UT-1 uLMS cells were treated with BET protein inhibitors JQ1 and I-BET 762 at a concentration of 5 µM for 48 h. Total RNA was isolated using Trizol. RNA quality and quantity were assessed using the Agilent bio-analyzer. The RNA-Seq library preparation and assessments of library quality and quantity were described previously [5]. An Illumina NovaSEQ6000 instrument was used for RNA sequencing.

### 2.8. Transcriptome Profiles Analysis

#### 2.8.1. Transcriptome Data Analysis

Transcriptome data analysis was carried out using a variety of R packages. The quality of reads was controlled using FastQC (http://www.bioinformatics.babraham.ac.uk/projects/fastqc (accessed on 21 May 2023), version 0.73, and then the reads were processed using Trimmomatic [36]. The version and parameters were chosen to trim the reads according to the FastQC results. The reads were mapped to the human reference genome, version hg38, using Hisat2 [37], version 2.2.1. Next, raw reads were mapped to the human reference transcriptome using feature counts [38], version 2.0.3, and the annotation file of Gencode [39], version V41. Gene counts were pre-processed and normalized using the DESeq2 [40] package in R, version 1.36.0. The quality of samples was examined using a comparative boxplot and PCA, and outlier samples were excluded from the analysis. Differentially expressed genes were identified using DESeq2.

#### 2.8.2. Functional Enrichment Analysis

Gene set enrichment analysis (GSEA) preranked [41] was performed using the fgsea R package [42], version 1.22.0, with gene set collections downloaded from the Molecular Signatures Database (MSigDB v7.5.1 for H (hallmark gene sets) and C2 (curated gene sets). The significant pathways were determined based on the parameters, including *n* = 1000 permutations, where *p*-adjust < 0.05, and FDR < 0.05. The Enrichplot (https://yulab-smu.top/biomedical-knowledge-mining-book (accessed on 21 May 2023) R package, version 1.16.2, was utilized to visualize the results. Additionally, we conducted histone modification enrichment analysis using ENCODE Histone Modifications 2015 in EnrichR [43] through clusterProfiler [44], version 4.4.4, to uncover the epigenetic mechanisms underlying the regulation of DEGs.

#### 2.8.3. Key Transcription Factor Identification

CeTF [45] R package, version 1.10.1, was used to identify the main TFs that control gene expression in different biological conditions, using Regulatory Impact Factors and Partial Correlation and Information Theory analysis. Co-expression networks were constructed using DEGs. To narrow down the TFs and acquire meaningful results, TFs with |FoldChange| > 1.5 and adjusted *p*-value < 0.05 in both JQ1 and I-BET 762 vs. control conditions were considered significant results.

#### 2.8.4. Survival Analysis of BRD Genes Expression in Sarcoma Patients

We utilized Gene Expression Profiling Interactive Analysis (GEPIA) (http://gepia.cancer-pku.cn/ (accessed on 29 June 2024) to conduct survival analysis, specifically examining overall survival (OS) in sarcoma patients. Patients were categorized into two groups based on the median expression levels of BRD2, BRD3, and BRD4 in sarcoma cancer samples, resulting in high and low expression groups. Subsequently, Kaplan–Meier methods were employed to generate survival curves. Additionally, we calculated the hazard ratio (HR) and *p*-value, with a *p*-value < 0.05 denoting statistical significance.

### 2.9. Statistical Analysis

Statistical analysis was performed as described previously [5]. A *p*-value of less than 0.05 was considered statistically significant.

## 3. Results

### 3.1. The BET Proteins BRD2, BRD3, and BRD4 Are Aberrantly Overexpressed in uLMS Tissues Compared to Adjacent Myometrium from Women with uLMS

**Figure 1:**
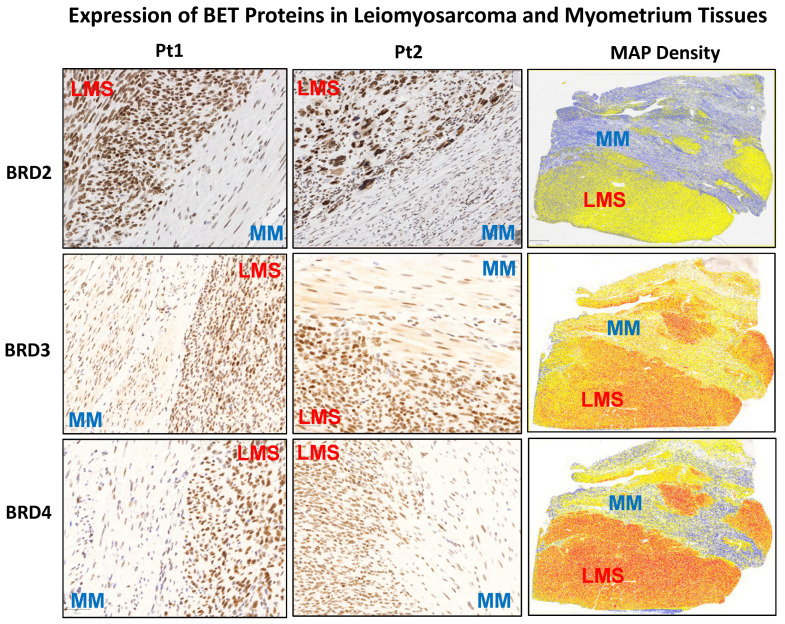
IHC staining of BRD2, 3, and 4 in human uLMS tissues and adjacent myometrium. IHC staining for BRD2, 3, and 4 is presented with two representative cases. The right column shows the density map of BRD2, 3, and 4 for the same representative case. Blue color: negative; yellow color: low expression; brown color: moderate expression; red color: strong expression. MM: myometrial tissues; LMS: leiomyosarcoma.

**Figure 2:**
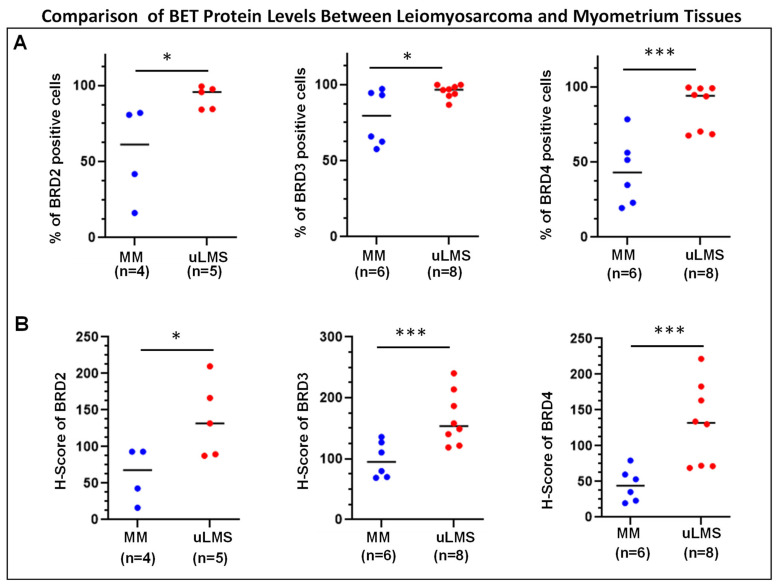
Percentage of BET protein-positive cells and H-score of BET protein expression in uLMS vs. myometrium. (**A**) Percentage of BRD2, BRD3, and BRD4 positive cells in uLMS and myometrium tissues. (**B**) H-score of BRD2, BRD3, and BRD4 in uLMS and myometrium tissues. * *p* < 0.05. *** *p* < 0.001.

To determine the differential levels of BET proteins between uLMS and MM (myometrium), IHC staining for BRD2, BRD3, and BRD4 was performed. Figure 1 and Figure 2 show that both staining intensity and the percentage of BRD2-, BRD3-, and BRD4-positive cells were significantly higher in uLMS than in MM. These studies reveal the dysregulation of BET protein expression in the pathogenesis and progression of uLMS.

### 3.2. Inhibition of BET BRDs Altered uLMS Cell Viability

**Figure 3:**
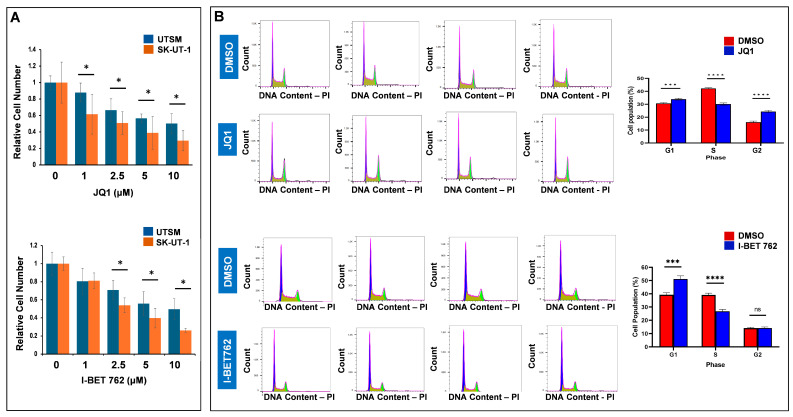
Treatment with JQ1 and I-BET 762 decreases uLMS cell viability and induces cell cycle arrest. (**A**) Cell viability of SK-UT-1 and UTSM cells were measured with trypan blue exclusion assay in the presence or absence of JQ1 and I-BET 762 for 48 h. (**B**) Treatment with JQ1 and I-BET 762 induces cell cycle arrest. Flow cytometric analysis was performed to measure the cell cycle phase distribution in SK-UT-1 uLMS cells in the presence or absence of JQ1 and I-BET 762. Cell cycle phases were marked as purple (G1), olive green (S), and light green (G2). Quantitative cell population analysis in response to JQ1 and I-BET 762 treatment was performed, respectively (right panel). DMSO group (*n* = 4), JQ1 (*n* = 4), I-BET 762 (*n* = 4). ns: no significant difference; * *p* < 0.05; *** *p* < 0.001; **** *p* < 0.0001. ns: no significant difference. n indicates the number of biological samples for each group.

Two potent and selective BET protein inhibitors, JQ1 and I-BET 762, were identified in 2010 [46,47,48]. Both cell-permeable small inhibitors can bind to the BRD pocket in a manner competitive with acetylated peptide binding [46,47]. Furthermore, both BET inhibitors engage the bromodomain pocket competitively with acetylated peptide binding, thereby causing the displacement of all four BET proteins from chromatin in cells upon exposure to these compounds [49]. Therefore, we selected JQ1 and I-BET 762 to assess their effects on uLMS cell viability. Cell viability was evaluated using a trypan blue exclusion assay in the SK-UT-1 cell line treated for 48 h with JQ1/I-BET 762 at 1–10 µM doese. Prolonged treatment (48 h) with JQ1/I-BET 762 showed a dose-dependent inhibitory effect on the viability of SK-UT-1 cells (Figure 3A). In contrast, there was much less inhibitory effect (compared with UTSM) on the control myometrial cell line (UTSM) (Figure 3A). These results indicate that JQ1 and I-BET 762 inhibit the UF cells in a dose-dependent manner and that it preferentially targets UF cells compared with UTSM cells.

### 3.3. Inhibition of BET BRDs Induces Cell Cycle Arrest in uLMS Cells

JQ1 treatment induced the accumulation of cells in the G1 phase and displayed a pronounced decrease of cells in the S phase, indicating the blockade of G1 progression (Figure 3B). The percentage of cells in the G1 phase increased from 30.6% to 33.9% (*p* < 0.0001) in response to 5 µM JQ1 treatment for 24 h. Accordingly, the percentage of cells in the S phase decreased from 42.3% to 30.2% (*p* < 0.0001) in response to the JQ1 treatment for 24 h. In addition, JQ1 treatment also significantly increased the cell population in the G2 phase. The percentage of cells in the G2 phase increased from 16.1% to 24.4% (*p* < 0.00001) in response to 5 µM JQ1 treatment (Figure 3B, right panel). I-BET 762 treatment also increased the accumulation of cells in the G1 phase and a corresponding decrease in the S phase, indicating the G1 progression blockade (Figure 3B). The percentage of cells in the G1 phase increased from 39.3% to 51.2% (*p* < 0.0001) in response to 5 µM I-BET 762 treatment. Accordingly, the percentage of cells in the S phase decreased from 39.2% to 26.8% (*p* < 0.0001) upon the I-BET 762 treatment (Figure 3B, right panel). These results suggest that the targeted inhibition of BET BRDs with JQ1 and I-BET 762 suppressed uLMS proliferation via cell cycle arrest (Figure 3A,B).

### 3.4. BET Protein Inhibition Causes Transcriptome Alterations in uLMS Cells

**Figure 4:**
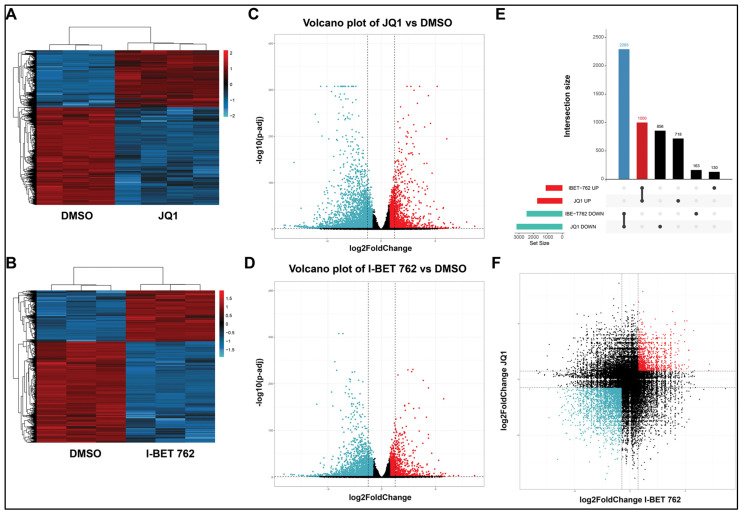
Treatments with JQ1 and I-BET 762 sculpt the transcriptome of uLMS cells. Heat maps are presented to cluster DEGs (JQ1 vs. control) (**A**) and (I-BET 762 vs. control) (**B**), respectively. Volcano plots of gene expression profiles are presented for JQ1 vs. control (**C**) and I-BET 762 vs. control (**D**). The red and blue points represent upregulated and downregulated genes, respectively. The vertical dotted and the horizontal black lines represent the log (FC) cutoff and the logarithmic transformed adjusted *p*-value cutoff, respectively. (**E**) Upset diagram showing the intersection size of upregulated and downregulated genes across drug treatments. (**F**) Distribution of overlapped DEGs in response to JQ1 and I-BET 762 treatments. FC: fold-change.

To examine the impact of BET protein inhibition on the uLMs transcriptome, we performed RNA-seq in SK-UT-1 cells treated without (DMSO control) or with JQ1 or I-BET 762 for 48 h. Differential gene expression analysis was done using three algorithms: limma-voom, DESeq2, and edgeR. Transcriptome analysis revealed that the treatment of SK-UT-1 cells with 5 µM JQ1 or I-BET-762 for 48 h induced 3586 DEGs (1130 up and 2456 down) and 4867 (1718 up and 3149 down), respectively. Figure 4A,B exhibit distinct expression patterns between the DMSO control group vs. JQ1 and I-BET 762 treatment groups, respectively. Figure 4C,D reveal the distribution of DEGs between treatment groups and the DMSO control group.

To visualize the intersections of DEGs in response to treatment with either JQ1 or I-BET 762, an upset plot was utilized to present the distribution characteristics of DEGs upon either drug treatment. As shown in Figure 4E, the common up and down DEGs with JQ1 and I-BET 762 contain 1000 and 2293 genes, respectively. The latter group exhibited the most significant number of genes in all groups involved in the two types of treatments. Figure 4F shows the distribution of unique and overlapped DEGs in response to JQ1 and I-BET 762 treatments.

**Figure 5:**
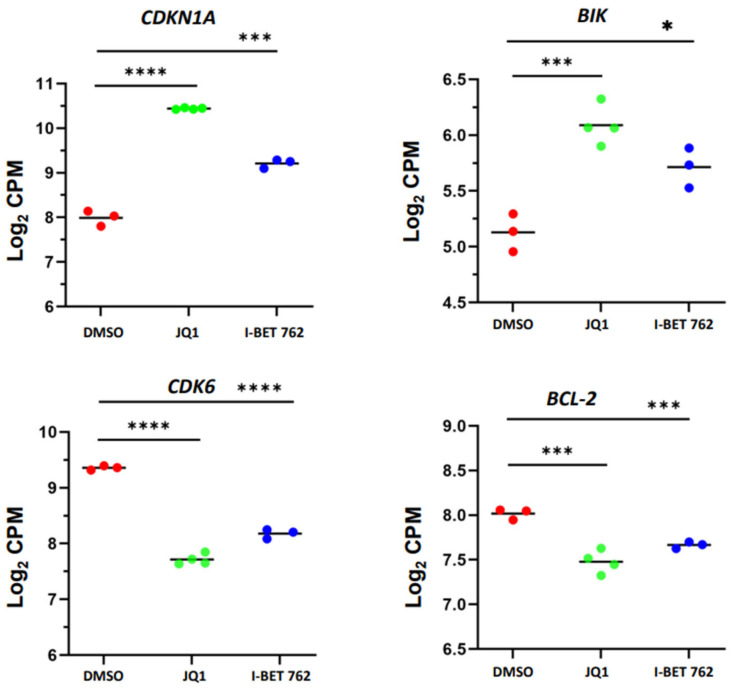
JQ1 and I-BET 762 altered cell cycle- and apoptosis-related gene expression in uLMS cells. RNA-seq analysis revealed the upregulation of *CDKN1A* and *BIK* and the downregulation of *CDK6* and *BCL2*, respectively, in uLMS cells upon BETis treatment. NS: no significant difference; * *p* < 0.05; *** *p* < 0.001; **** *p* < 0.0001.

To determine if our RNA-seq data correlates with biological effects impacted by BETis, we initially compared the expression levels of cell cycle-related genes between control and BETi-treated uLMS cells. As shown in Figure 5, JQ1 and I-BET 762 treatments increased the expression levels of *CDKN1A* and reduced the expression levels of *CDK6*, which correlated with our finding that BETis induced cell cycle arrest. In addition, BETis induced the expression of BIK, which can stimulate apoptosis, while decreasing the expression of BCL-2, which can suppress apoptosis. Our results suggested that these cell progression regulators may be critical in BETi1-induced cell cycle arrest.

### 3.5. Pathway Analysis of DEGs upon JQ1 and I-BET 762 Treatments

**Figure 6:**
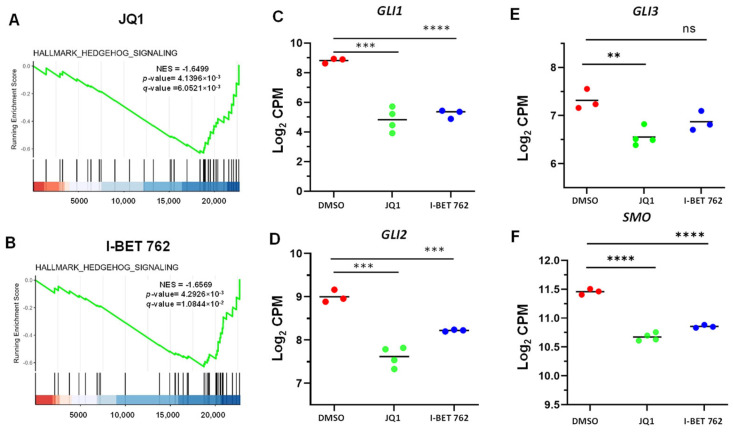
JQ1 and I-BET 762 altered the Hedgehog pathway in uLMS cells. Hallmark analysis demonstrated the enrichment of the Hedgehog pathway in SK₋UT₋1 cells in response to JQ1 (**A**) and I-BET 762 (**B**) treatments. The key components of the Hedgehog pathway, including *GLI1* (**C**), *GLI2* (**D**), *GLI3* (**E**), and *SMO* (**F**), are downregulated in response to JQ1 and I-BET 762 treatment. ** *p* < 0.01; *** *p* < 0.001; **** *p* < 0.0001. ns: no significant difference.

To gain insight into the biological changes by BET protein inhibition, gene set enrichment analysis (GSEA) was performed. We demonstrated that Hedgehog signaling was suppressed in response to JQ1 and I-BET 762 treatments, as shown in Figure 6A,B. Accordingly, several key components of the Hedgehog signaling pathway, including GL1, 2, 3, and SMO, were downregulated in BETi-treated uLMS cells compared to control cells treated with the vehicle (DMSO) (Figure 6C–F).

**Figure 7:**
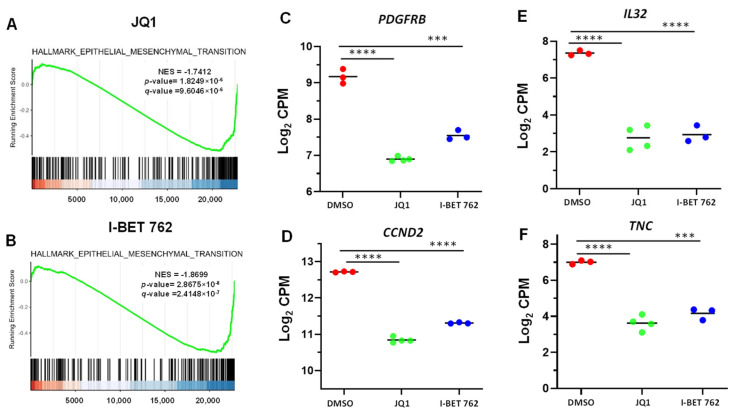
JQ1 and I-BET 762 altered the EMT pathway in uLMS cells. Hallmark analysis demonstrated the enrichment of the EMT pathway in SK₋UT₋1 cells in response to JQ1 (**A**) and I-BET 762 (**B**) treatments. The EMT inducers, including *PDGFRβ* (**C**), *CCND2* (**D**), *IL32* (**E**), and *TNC* (**F**), are downregulated in response to BETi treatment. *** *p* < 0.001; **** *p* < 0.0001.

In addition, we demonstrated that epithelial–mesenchymal transition (EMT) was inhibited in response to BETi treatment in uLMS cells (Figure 7A,B). Accordingly, EMT inducers, including PDGFRβ, CCND2, IL32, and TNC, were downregulated in uLMS cells treated with BETis (Figure 7C–F).

In addition to Hedgehog signaling and EMT, JQ1 and I-BET762 altered other key pathways, including metastasis, invasive cancer, and cell migration (Figure S2).

**Figure 8:**
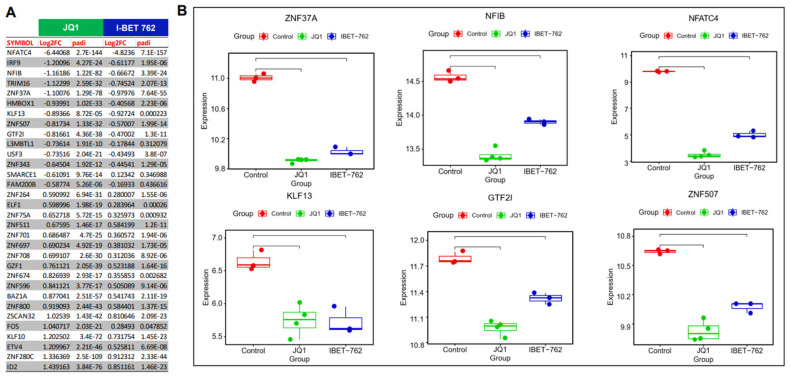
Targeted inhibition of BET proteins suppressed the gene expression of TFs, contributing to cancer metastasis and progression. (**A**) The list of DEGs of TFs (**B**) RNA-seq revealed the downregulation of TF genes, including *ZNF37A*, *NFIB*, *NFATC4*, *KLF13*, *GTF2I*, and *ZNF507* in uLMS cells.

TFs play a critical role in cancer progression. Therefore, we determined the TFs involved in the metastasis of uLMS. We identified several TFs, including GTF2I, NFIB, NFATC4, ZNF37A, KLF13, and ZNF507, involved in cancer metastasis. BETis decreased the expression of these TFs in uLMS cells. Therefore, BETis may reduce the metastasis of uLMS by inhibiting the metastasis-related TFs (Figure 8).

### 3.6. JQ1 and I-BET 762 Treatment Altered the Expression of Epigenetic Regulators

**Figure 9:**
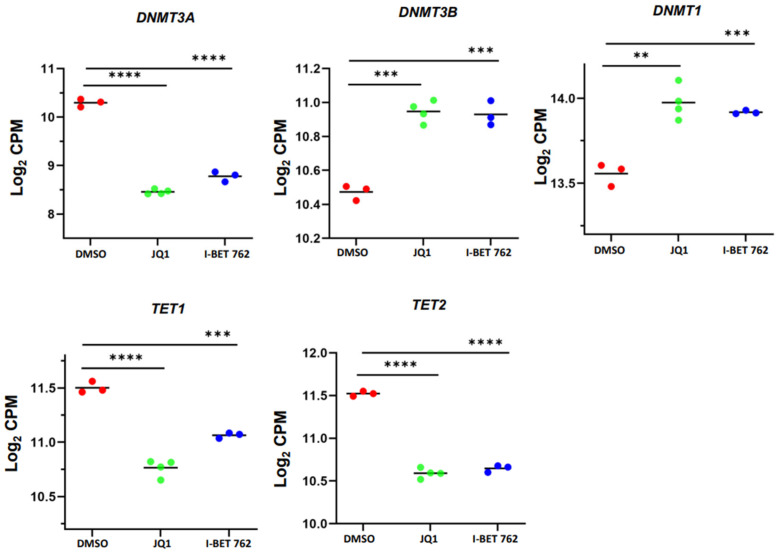
JQ1 and I-BET 762 treatments altered DNA methylation-related genes in uLMS cells. RNA−seq revealed the downregulation of *DNMT3A*, *DNMT3B*, *DNMT1*, *TET1*, and *TET2* in uLMS cells in response to JQ1 and I-BET 762 treatment. ** *p* < 0.01; *** *p* < 0.001; **** *p* < 0.0001.

Previous studies identified the functional link between DNA methylation and chromatin modifications [50,51,52]. In this study, we investigated whether inhibiting BET proteins altered the expression levels of DNA methylation regulators. Accordingly, we employed a targeted gene analysis approach and revealed that the expression levels of genes that regulated the dynamic status of DNA methylation were modulated in BETi-treated SK-UT-1 cells. These DNA methylation/demethylation-related DEGs included *DNMT3A*, *DNMT3B*, *DNMT1*, *TET1*, and *TET2* (Figure 9).

**Figure 10:**
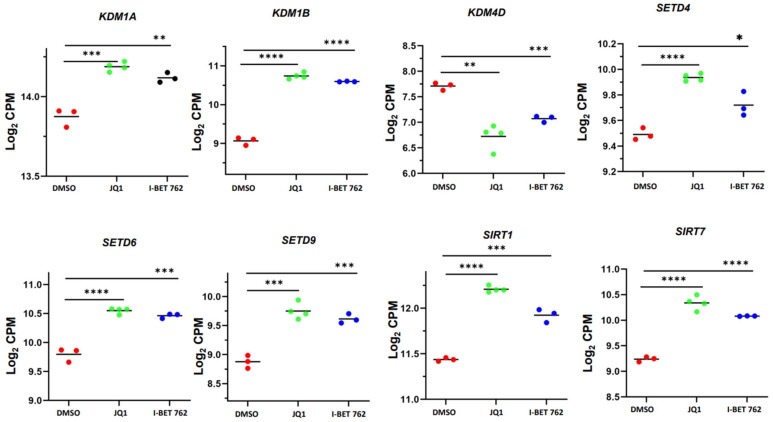
JQ1 and I-BET 762 altered the expression levels of histone modification-regulated genes in uLMS cells. RNA−seq revealed the altered expression of *KDM1A*, *KDM1B*, *KDM4D*, *SETD6*, *SETD9*, *SETDB1*, *SET*, *SETD9*, *SIRT1*, and *SIRT7* in uLMS cells in response to BETi treatments. * *p* < 0.05; ** *p* < 0.01; *** *p* < 0.001; **** *p* < 0.0001.

To determine the relationship between chromatin readers and histone modifications, we characterized the genes related to histone modifications in SK-UT-1 cells after treatment with BETis. As shown in Figure 10, the targeted inhibition of BET proteins by JQ1 and I-BET 762 significantly modulated the expression levels of *KDM1A*, *KDM1B*, *KDM4D*, *SETD4*, *SETD6*, *SETD9*, *SIRT1*, and *SIRT7.* These analyses suggest that JQ1 and I-BET 762 treatments may alter the transcriptome via histone modifications.

To better understand epigenetic-regulated transcriptional changes in response to the JQ1 and I-BET 762 treatment, we performed an enrichment analysis of epigenetic histone markers using the Enrichr web server for our further discoveries. As shown in Figure S3, we identified several histone modifications, including H4K20me1 and H3K4me3, associated with upregulated DEGs in response to JQ1 treatment (Figure S3A). In addition, we identified downregulated DEGs related to histone modifications, including H3K27me3 (Figure S3B). For the association analysis between I-BET 762-induced DEGs and histone marks, we revealed that histone modifications with up DEGs included H4K20me1 and H3K4me3, among others (Figure S3A, right panel). The histone modifications with I-BET 762-induced down DEGs included H3K27me3 (Figure S3B, right panel).

### 3.7. JQ1 and I-BET 762 Treatment Altered the Expression of m^6^A Regulators

**Figure 11:**
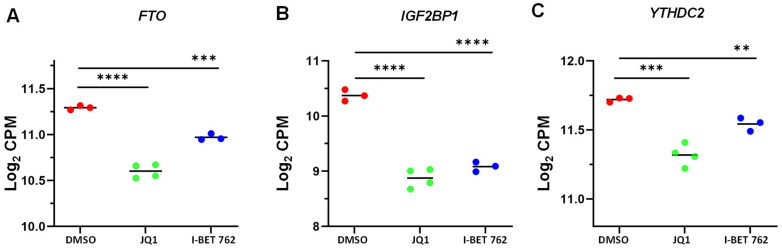
JQ1 and I-BET 762 altered the expression levels of m^6^A regulators in uLMS cells. RNA−seq revealed the altered expression of *FTO* (**A**), *IGF2BP1* (**B**), and *YTHDC2* (**C**) in uLMS cells in response to BETi treatment. ** *p* < 0.01; *** *p* < 0.001; **** *p* < 0.0001.

To determine the impact of BET protein inhibition on RNA epigenetics, we compared the expression of several key m^6^A regulators between control and BETi-treated groups. As shown in Figure 11, JQ1 and I-BET 762 altered the expression levels of *FTO*, *YTHDC2*, and *IGF2BP1*, indicating that BET proteins may participate in reprogramming the m^6^A epitranscriptome in uLMS.

To validate the DEGs related to the cell cycle, Hedgehog pathway, EMT, TF-driven pathway, and epigenetic regulators, we selected several key genes and performed RT-qPCR analysis to confirm our findings. As shown in Figure S4A–D, the expression levels of genes related to the aforementioned biological progress between control and JQ1/I-BET 762 treated cells are consistent with RNA-seq data.

### 3.8. The Impact of BET BRD Gene Expression on Survival Rates in Sarcoma Patients

**Figure 12:**
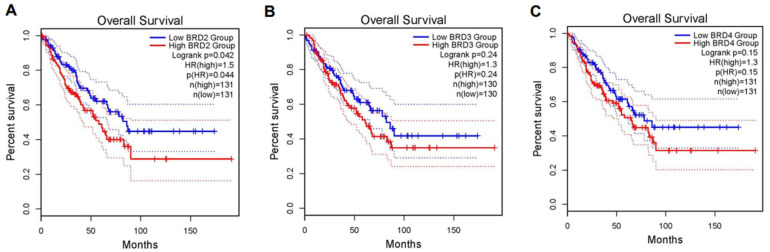
Kaplan–Meier curves of sarcoma patient’s OS with low BRD expression (blue) versus high gene expression (red line). (**A**) BRD2; (**B**) BRD3; (**C**) BRD4. Human data from the TCGA dataset were accessed. The patients were categorized with expression levels above the median as the high group (n = 131) and those below the median as the low group (n = 131).

This study demonstrated that BET proteins, BRD2, BRD3, and BRD4, are aberrantly overexpressed in uLMS tissues compared to the adjacent myometrium. Therefore, we investigated if the BRD protein expression levels are correlated with the OS of sarcoma patients. Our survival analysis revealed a significant difference in OS between the high (n = 131) and low expression (n = 131) groups of the BRD2 gene in sarcoma patients. Patients with higher expression levels of BRD2 had a significantly (*p*-value < 0.05) lower survival rate (1.5-fold) compared to those with lower expression levels (Figure 12A). The Kaplan–Meier survival curves demonstrated this disparity, with a calculated hazard ratio (HR) indicating a higher mortality risk in the high-expression group. The analysis for BRD3 and BRD4 gene expression exhibited a similar trend compared to BRD2 expression in terms of OS; however, the expression levels of BRD3 and BRD4 did not show a significant impact on the OS of uLMS patients (Figure 12B,C).

## 4. Discussion

uLMS is a highly aggressive tumor type with a high tumor recurrence rate, progression, and metastasis [4]. The origin and molecular mechanism underlying and driving its clinical and biological behavior remain unclear. Although the role of epigenetic alteration in uLMS has been investigated, the functional role and epigenetic mechanism underlying BET protein-related uLMS pathogenesis are limited. In this study, we determined the expression pattern of BET proteins in uLMS tissues and examined the biological effect of the targeted inhibition of BET proteins in uLMS cells. We demonstrated that BET proteins, including BRD2, 3, and 4, were aberrantly upregulated in uLMS and exhibited an important tumor-promoting role in uLMS. Accordingly, we utilized GEPIA to conduct survival analysis and demonstrated that BRD2 expression levels were significantly associated with the OS of sarcoma patients. Furthermore, BET proteins could alter several key pathways and reprogram the oncogenome in uLMS. In addition, specific BET protein inhibitors show promising therapeutic efficacy in treating uLMS.

The aberrant overexpression of BET proteins has been found in many cancer types and often correlates with the cancer phenotype. For instance, BRD4 expression was upregulated in pediatric primary medulloblastomas [53] and was found to be overexpressed in ovarian cancer, which is correlated with BRD4 amplification [23]. Notably, BET proteins as a therapeutic target have been reported in many cancers, including gynecological ovarian cancer [23,54,55,56]. We previously reported that uLMS cells grow faster than myometrial cells [57]. This study demonstrated that inhibiting BET proteins with JQ1 and I-BET 762 significantly decreased uLMS proliferation dose-dependently via cell cycle arrest. Other reports showed that JQ1 could reduce cancer cell growth in vitro and in vivo [58,59]. The underlying mechanisms include an effect of BETis on cell cycle arrest in the G1 phase and a decrease in the percentage of cells in the S phase. Accordingly, several BET protein inhibitors have been developed, and these pharmacologic inhibitors showed potent anti-tumor effects with decreased cancer phenotypes via multiple mechanisms dependent upon the cancer types and experimental conditions [60]. For example, treating medulloblastoma cell lines with JQ1 significantly decreased cell proliferation and preferentially induced apoptosis in *MYC*-overexpressed cells. Additionally, JQ1 treatment prolonged the survival of mice harboring medulloblastoma xenografts and diminished the tumor burden in these mice [53]. Integrating of genetic features with chemosensitivity data revealed a robust correlation between MYCN amplification and sensitivity to bromodomain inhibition in pediatric cancer neuroblastoma. BET inhibition conferred a significant survival advantage in neuroblastoma models via regulating MYCN, providing a compelling rationale for developing BET bromodomain inhibitors in patients with neuroblastoma [61]. In addition, BET inhibitors also provide multiple beneficial effects on anti-inflammation [47], heart failure [62], spermatogenesis [63], and chemoresistance [64,65].

To further determine the mechanisms associated with JQ1/I-BET 762-induced inhibition, we performed a genome-wide RNA-sequencing analysis comparing the profiles of DMSO-treated with BETi-treated uLMS cells. Our high-throughput sequencing analysis revealed that the targeted inhibition of BET proteins with BETis altered several critical biological pathways that may contribute to uLMS pathogenesis. Hedgehog signaling plays a fundamental role in several biological processes, including embryonic development, tissue repair, the proliferation and differentiation of various cells, hematopoiesis, as well as the pathogenesis of various types of cancer [66,67,68,69,70]. In this study, we demonstrated that BETi altered the Hedgehog pathway in uLMS. Furthermore, several key components in the Hedgehog pathway, including GLI1, GLI2, SMO, etc., were downregulated in response to BETi treatment. We have previously reported that the Hedgehog pathway was activated in uLMS with aberrant upregulation of the GLI family and increased nuclear translocation of GLI1 [70]. Therefore, BET inhibitors may suppress the uLMS phenotype by inhibiting the Hedgehog pathway. Interestingly, CCND2, an established GLL1 target gene [71], is also decreased in BETi-treated uLMS cells. In addition to an in vitro study on the role of BRD “reader” inhibition in LMS, a BET bromodomain inhibitor, GS-626510, has been tested in the LMS patient-derived xenograft model harboring either derangements in C-MYC and PTEN/PI3CA/AKT genes or homologous recombination deficiency signatures. The study demonstrated that GS-626510, a BET family BRD inhibitor, suppressed the LMS tumor growth in these two models [72]. These studies elucidate the critical role of histone readers in the pathogenesis of LMS (Figure S3).

EMT is a biological process crucial for tumor aggressiveness, including cancer invasion and metastasis as well as drug response [73,74]. Therefore, the cellular EMT status can be considered a reliable determinant of patient prognosis. In this study, we demonstrated that the targeted inhibition of BET proteins decreased the EMT pathway in uLMS cells. We have identified several EMT inducers in uLMS cells with a significantly decreased expression in response to BETi treatment, including *IL32*, *TNC*, and *PDGFRβ*. IL32 is a cytokine that plays a fundamental role in innate and acquired immunity through the regulation of T cells [75,76]. IL32 can trigger the onset of ECM in several types of cells [75,77]. Tenascin-C (TNC) is a large extracellular matrix glycoprotein that promotes cell adhesion and tissue remodeling and plays a critical role in the transduction of cellular signaling pathways [78]. TNC promotes EMT and relevant pathways in several types of cancer [78,79,80]. The inhibition of TNC by knockdown can inhibit cancer cell proliferation, migration, and invasion and suppress tumor growth in vivo [78]. PDGFRβ belongs to the type III receptor tyrosine kinase family and is known to be involved in tumor metastasis [81,82]. In addition, PDGFRβ promotes the EMT process via the activation of the PI3K/ERK pathway [83,84]. All these data suggested that BET inhibitors suppressed the uLMS phenotype by inhibiting the EMT pathway.

TFs play a central role in cancer progression by modulating the interplay between cell signaling and gene regulation. Some TFs alter multiple biological processes, including differentiation and development, DNA repair genes, cell proliferation, cellular stresses, and therapy resistance. Beyond these roles, various TFs also regulate cancer invasion, metastasis, and progression [85,86,87]. TFs, often in conjunction with their corresponding co-activators or co-repressors, can cause alterations in gene expression at specific genome sites [88]. In this study, we identified multiple TFs involved in cancer metastasis. For instance, ZNF37A has been found to be involved in promoting tumor metastasis via the THSD4/TGF-β axis in colorectal cancer [89]. In prostate cancer (PC), ZNF507 expression was associated with metastatic PC with a high grade. Furthermore, ZNF507 promoted the metastatic properties of PC by enhancing TGF-β signaling [90]. GTF21, as a transcriptional factor, binds to the initiator and E-box element in promoters and modulates gene expression. It is reported that GTF2I was found to have potential prognostic value for breast cancer metastasis [91]. Integrated analysis demonstrated that KLF13 may be involved in tumorigenesis and metastasis in colon cancer [92]. NFIB has been reported to promote tumor growth, metastasis, and recurrence in various cancers [93]. Another TF, NFATC4, has been reported to be aberrantly activated and is involved in initiation, proliferation, invasion, and metastasis in several types of cancer [94]. Altogether, BET inhibitors may suppress EMT via TF alterations in uLMS.

Notably, BET proteins play a central role in gene transcription in chromatin [95]. The interplay between BET proteins and chromatin regulation and organization has been reported [96]. A recent study by Zhou et al. demonstrated that BRD4 was highly expressed in gastric cancer (GC) tissues and was significantly associated with poor prognosis. JQ1 inhibits the malignant progression of GC by downregulating chromatin accessibility and inactivating RUNX2/NID1 signaling [96]. In our loss-of-function study, we demonstrated that the targeted inhibition of BET proteins altered the expression levels of histone acetylation modulators, histone methylation enzymes, and DNA-methylation-related epigenetic regulators, further demonstrating the substantial crosstalk between BET proteins and other epigenetic mechanisms. Accordingly, the targeted inhibition of BET proteins might alter the transcriptome by reprogramming the network of oncogenic epigenomes in uLMS.

N6-methyladenosine (m^6^A) is the most prevalent, abundant, and conserved posttranscriptional modification in eukaryotic RNAs and plays an important role in many biological processes [97]. Recently, we reported that m^6^A demethylase FTO plays an oncogenic role in the pathogenesis of uLMS [98]. Herein, we determined the link between BET proteins and RNA methylation regulators. Our study demonstrated that BETi decreased the expression of FTO m^6^A RNA demethylase. In addition, BETi also altered the expression of m^6^A readers, such as YTHDC2 and IGF2BP1. These data suggest that the targeted inhibition of BET proteins may alter the epitranscriptome in uLMS, emphasizing the importance of m^6^A regulation in uLMS progression.

**Figure 13:**
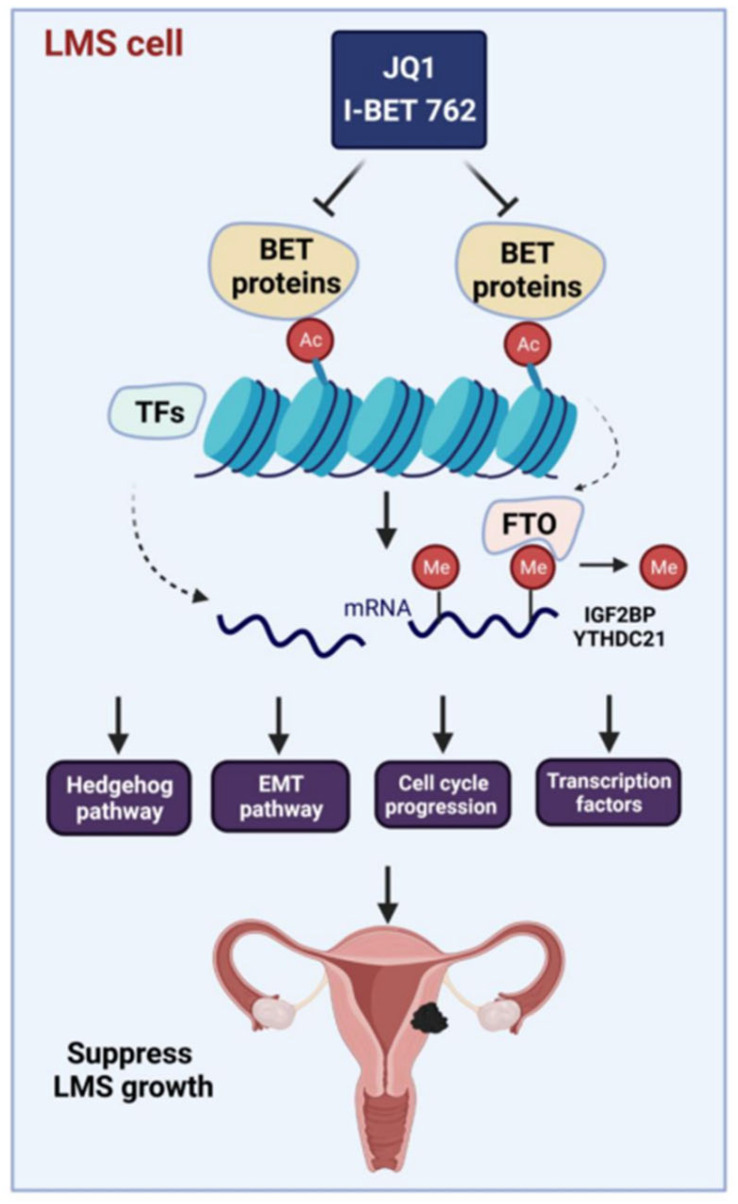
Experimental model. Our experimental model shows that targeting BET proteins with JQ1 and I-BET 762 induces cell cycle arrest, modulates the Hedgehog and EMT pathways, and alters the TF network as well as interactions between target genes and epigenetic regulators in uLMS cells. This figure was created using the BioRender software online app (BioRender.com).

Based on our studies, we propose a mechanistic model for the targeted inhibition of BET proteins in uLMS: (1) BET proteins, including BRD2, 3, and 4 are aberrantly overexpressed in uLMS compared to the adjacent myometrium; (2) targeting BET proteins with JQ1 and I-BET 762 alters the uLMS phenotype by suppressing cell proliferation via cell cycle arrest and the modulation of cell cycle-related genes and others; (3) BETis reversed the phenotype of uLMS via different biological pathways including the Hedgehog pathway, the EMT pathway, and TF-driven signaling; (4) BET proteins constitute a potential therapeutic vulnerability in malignant uLMS, and BET protein inhibitors, such as JQ1 and I-BET 762, alter key pathways and reprogram the oncogenic profiling and epigenetic network to suppress the uLMS phenotype (Figure 13).

## 5. Conclusions

In conclusion, our study demonstrated for the first time that uLMS tumors exhibited an aberrant upregulation of BET proteins, highlighting the critical role of histone readers in the pathogenesis of uLMS. The targeted inhibition of BET proteins may impart beneficial effects in uLMS and provide a promising and novel strategy for treating patients with this aggressive uterine cancer.

## Data Availability

Raw FASTQ files were deposited in the NCBI Gene Expression Omnibus (GSE275087).

## Supplementary Materials

The following supporting information can be downloaded at: https://www.mdpi.com/article/10.3390/cells13171443/s1, Figure S1: Flowchart of experimental design and bioinformatics analysis; Figure S2: Hallmark analysis demonstrated the enrichment of metastasis, invasive cancer, and cell migration in SK₋UT₋1 cells in response to JQ1 and I-BET 762 treatment; Figure S3: Targeted inhibition of BET proteins altered the gene expression correlating to histone modifications; Figure S4: The validation of differentially expressed genes between control (DMSO) and BETi-treated uLMS cells. Table S1: Primers used in the study.
