# Supplementary material for: Unraveling the Role of Bromodomain and Extra-Terminal Proteins in Human Uterine Leiomyosarcoma
Source: Cells. 2024 Aug 28;13(17):1443. doi: 10.3390/cells13171443 (PMC11394028; doi:10.3390/cells13171443)
Supplement: Supplementary file 1 [file cells-13-01443-s001.zip › cells-3119477-supplementary.pdf]

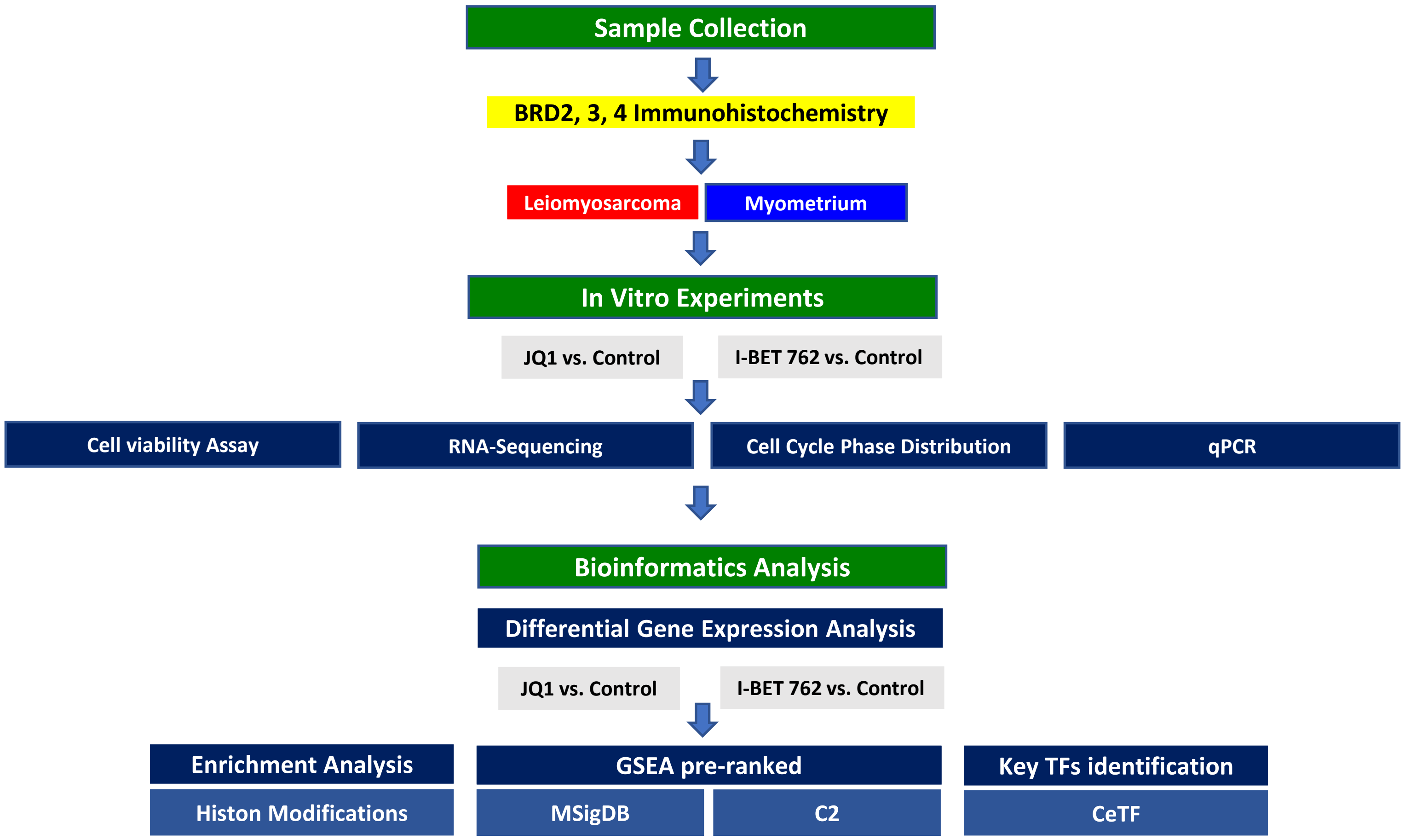
Sample Collection
BRD2, 3, 4 Immunohistochemistry
Leiomyosarcoma
Myometrium
In Vitro Experiments
JQ1 vs. Control
I-BET 762 vs. Control
Cell viability Assay
RNA-Sequencing
Cell Cycle Phase Distribution
qPCR
Bioinformatics Analysis
Differential Gene Expression Analysis
JQ1 vs. Control
I-BET 762 vs. Control
Enrichment Analysis
GSEA pre-ranked
Key TFs identification
Histon Modifications
MSigDB
C2
CeTF

Fig.S1

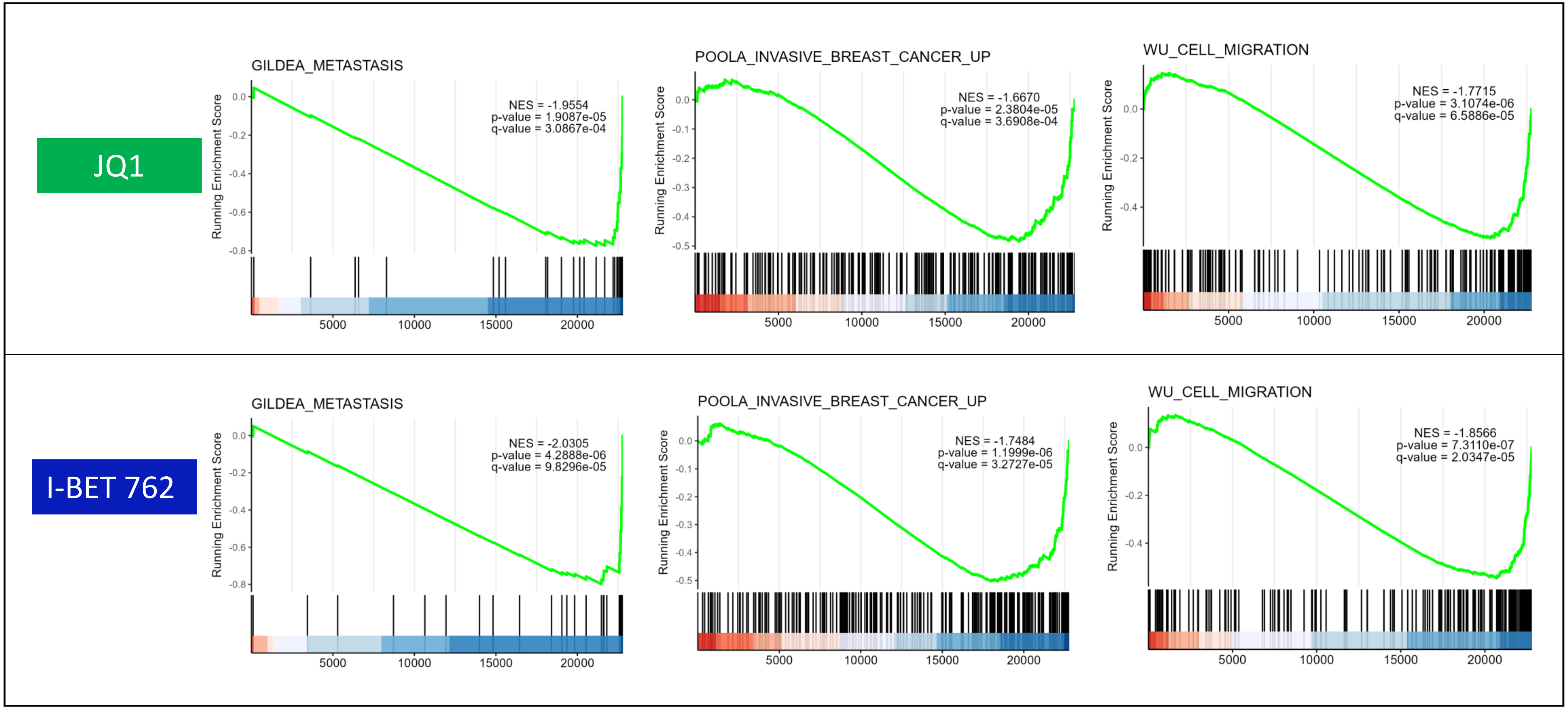
JQ1
GILDEA_METASTASIS
NES = -1.9554
p-value = 1.9087e-05
q-value = 3.0867e-04
POOLA_INVASIVE_BREAST_CANCER_UP
NES = -1.6670
p-value = 2.3804e-05
q-value = 3.6908e-04
WU_CELL_MIGRATION
NES = -1.7715
p-value = 3.1074e-06
q-value = 6.5886e-05
I-BET 762
GILDEA_METASTASIS
NES = -2.0305
p-value = 4.2888e-06
q-value = 9.8296e-05
POOLA_INVASIVE_BREAST_CANCER_UP
NES = -1.7484
p-value = 1.1999e-06
q-value = 3.2727e-05
WU_CELL_MIGRATION
NES = -1.8566
p-value = 7.3110e-07
q-value = 2.0347e-05
Running Enrichment Score

Fig.S2

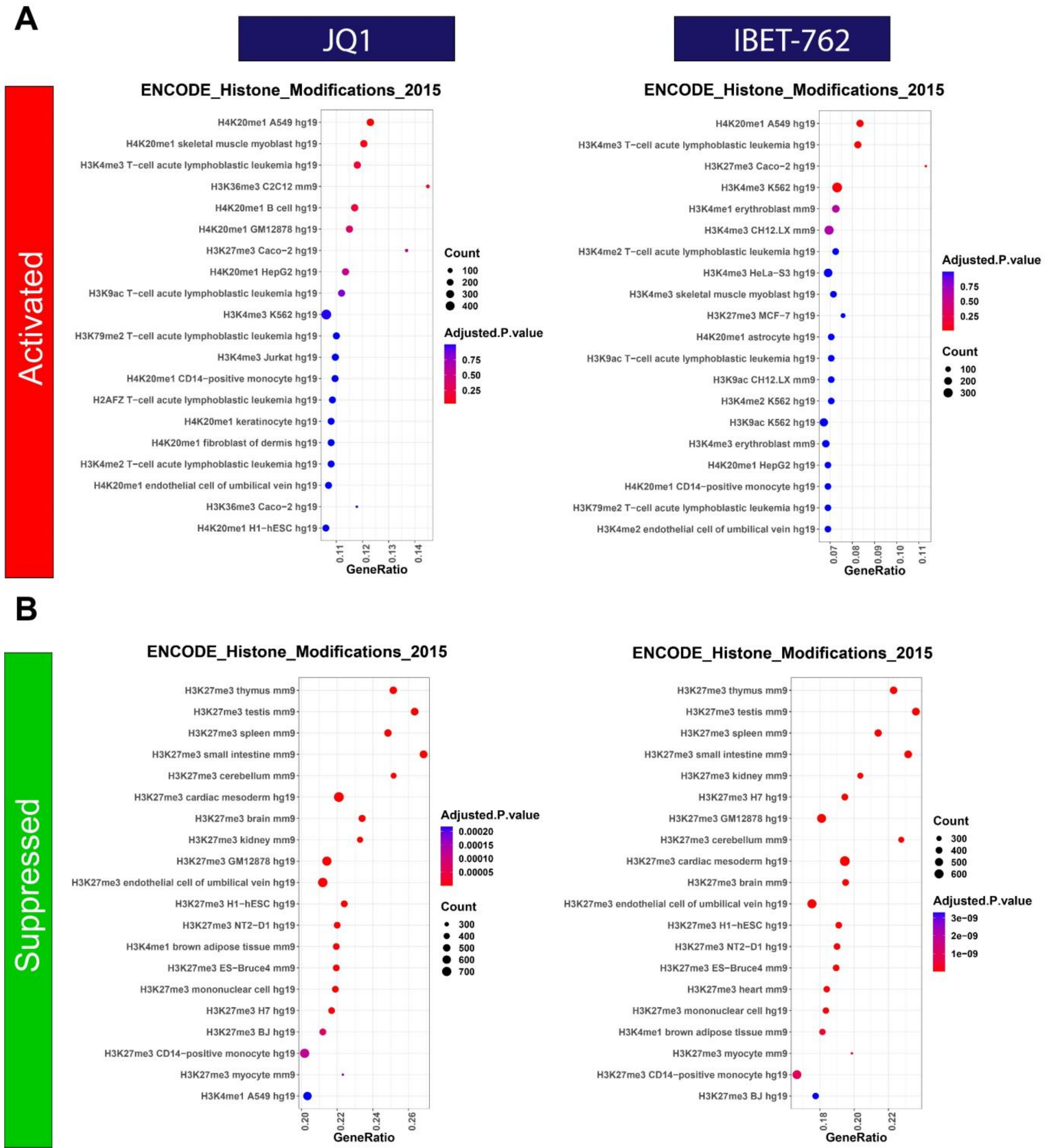
A
JQ1
IBET-762
Activated
ENCODE_Histone_Modifications_2015
H4K20me1 A549 hg19
H4K20me1 skeletal muscle myoblast hg19
H3K4me3 T-cell acute lymphoblastic leukemia hg19
H3K36me3 C2C12 mm9
H4K20me1 B cell hg19
H4K20me1 GM12878 hg19
H3K27me3 Caco-2 hg19
H4K20me1 HepG2 hg19
H3K9ac T-cell acute lymphoblastic leukemia hg19
H3K4me3 K562 hg19
H3K79me2 T-cell acute lymphoblastic leukemia hg19
H3K4me3 Jurkat hg19
H4K20me1 CD14-positive monocyte hg19
H2AFZ T-cell acute lymphoblastic leukemia hg19
H4K20me1 keratinocyte hg19
H4K20me1 fibroblast of dermis hg19
H3K4me2 T-cell acute lymphoblastic leukemia hg19
H4K20me1 endothelial cell of umbilical vein hg19
H3K36me3 Caco-2 hg19
H4K20me1 H1-hESC hg19
GeneRatio
Count
Adjusted.P.value
ENCODE_Histone_Modifications_2015
H4K20me1 A549 hg19
H3K4me3 T-cell acute lymphoblastic leukemia hg19
H3K27me3 Caco-2 hg19
H3K4me3 K562 hg19
H3K4me1 erythroblast mm9
H3K4me3 CH12.LX mm9
H3K4me2 T-cell acute lymphoblastic leukemia hg19
H3K4me3 HeLa-S3 hg19
H3K4me3 skeletal muscle myoblast hg19
H3K27me3 MCF-7 hg19
H4K20me1 astrocyte hg19
H3K9ac T-cell acute lymphoblastic leukemia hg19
H3K9ac CH12.LX mm9
H3K4me2 K562 hg19
H3K9ac K562 hg19
H3K4me3 erythroblast mm9
H4K20me1 HepG2 hg19
H4K20me1 CD14-positive monocyte hg19
H3K79me2 T-cell acute lymphoblastic leukemia hg19
H3K4me2 endothelial cell of umbilical vein hg19
GeneRatio
Adjusted.P.value
Count
B
Suppressed
ENCODE_Histone_Modifications_2015
H3K27me3 thymus mm9
H3K27me3 testis mm9
H3K27me3 spleen mm9
H3K27me3 small intestine mm9
H3K27me3 cerebellum mm9
H3K27me3 cardiac mesoderm hg19
H3K27me3 brain mm9
H3K27me3 kidney mm9
H3K27me3 GM12878 hg19
H3K27me3 endothelial cell of umbilical vein hg19
H3K27me3 H1-hESC hg19
H3K27me3 NT2-D1 hg19
H3K4me1 brown adipose tissue mm9
H3K27me3 ES-Bruce4 mm9
H3K27me3 mononuclear cell hg19
H3K27me3 H7 hg19
H3K27me3 BJ hg19
H3K27me3 CD14-positive monocyte hg19
H3K27me3 myocyte mm9
H3K4me1 A549 hg19
GeneRatio
Adjusted.P.value
Count
ENCODE_Histone_Modifications_2015
H3K27me3 thymus mm9
H3K27me3 testis mm9
H3K27me3 spleen mm9
H3K27me3 small intestine mm9
H3K27me3 kidney mm9
H3K27me3 H7 hg19
H3K27me3 GM12878 hg19
H3K27me3 cerebellum mm9
H3K27me3 cardiac mesoderm hg19
H3K27me3 brain mm9
H3K27me3 endothelial cell of umbilical vein hg19
H3K27me3 H1-hESC hg19
H3K27me3 NT2-D1 hg19
H3K27me3 ES-Bruce4 mm9
H3K27me3 heart mm9
H3K27me3 mononuclear cell hg19
H3K4me1 brown adipose tissue mm9
H3K27me3 myocyte mm9
H3K27me3 CD14-positive monocyte hg19
H3K27me3 BJ hg19
GeneRatio
Count
Adjusted.P.value

Fig.S3

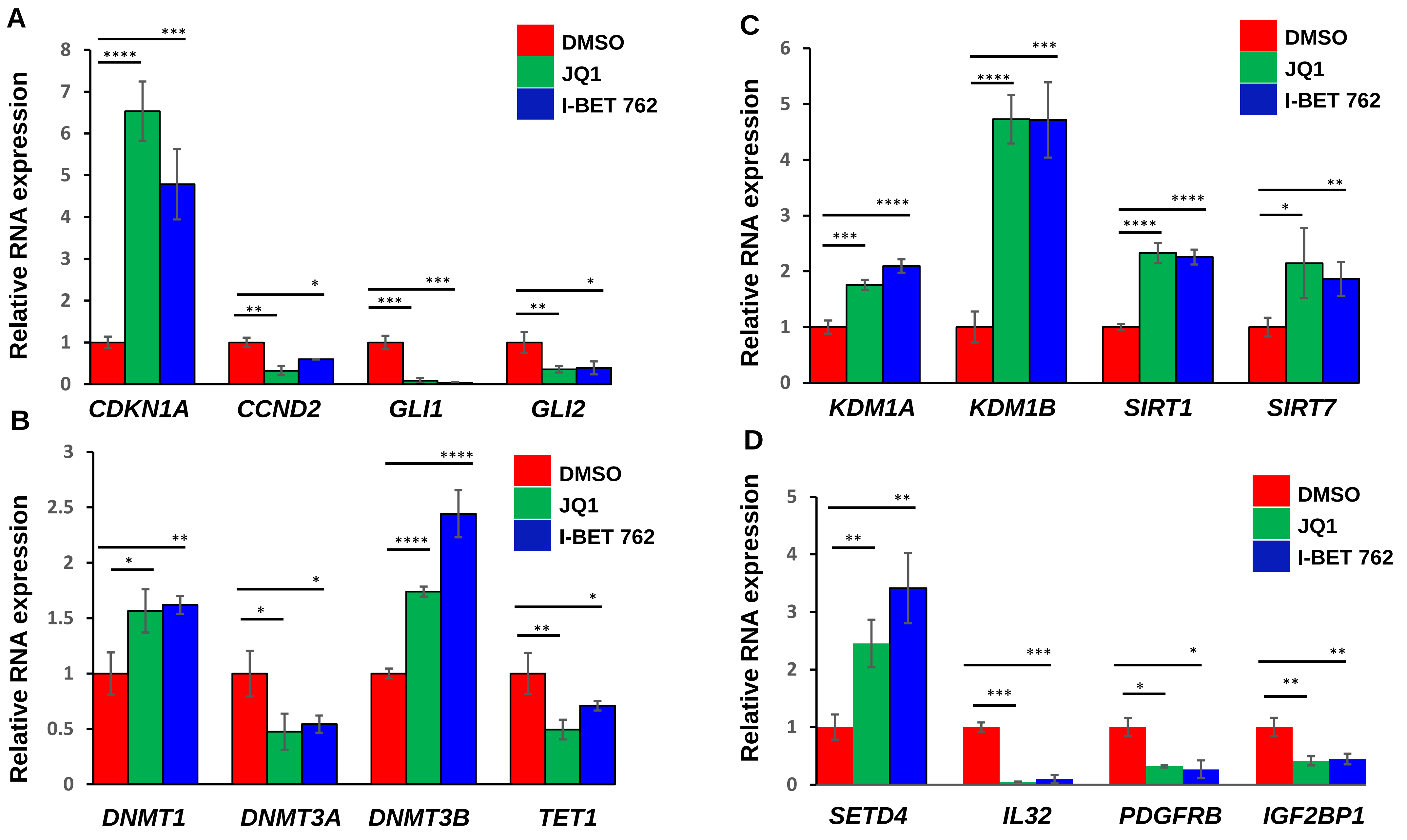
A
B
C
D
Relative RNA expression
DMSO
JQ1
I-BET 762
CDKN1A
CCND2
GLI1
GLI2
DNMT1
DNMT3A
DNMT3B
TET1
KDM1A
KDM1B
SIRT1
SIRT7
SETD4
IL32
PDGFRB
IGF2BP1

Fig.S4

Supplemental figure legends

Figure S1. Flowchart of experimental design and bioinformatics analysis

Figure S2. Hallmark analysis demonstrated the enrichment of metastasis, invasive cancer, and cell migration in SK-UT-1 cells in response to JQ1 and I-BET 762 treatment.

Figure S3. Targeted inhibition of BET proteins altered the gene expression correlating to histone modifications. The dot plots showed the top twenty enrichment terms for histone modification associated with up DEGs (A) and down DEGs (B) in response to JQ1 treatment. (C) histone modifications related to up DEGs in response to I-BET 762 treatment. (D) histone modifications associated with down DEGs in response to I-BET 762 treatment. The *X*-axis represents the gene ratio, and the *y*-axis describes the enrichment components. The area of the circle is proportional to the number of genes assigned to the term, and the color accords with the Adjusted *P*-value.

Figure S4. The validation of differentially expressed genes between control (DMSO) and BETi-treated uLMS cells. RNAs isolated from three groups (DMSO, JQ1, and I-BET 762) used for RNA-seq were subjected to cDNA synthesis and q-PCR analysis described in the methods. (A) The comparison of expression levels of genes associated with the cell cycle and hedgehog pathway between control and BETi-treated uLMS cells. (B) The comparison of expression levels of genes related to the dynamic regulation of DNA methylation between control and BETi-treated uLMS cells. (C) The comparison of expression levels of genes related to histone modifications between control and BETi-treated uLMS cells. (D) The comparison of expression levels of genes of *SETD4*, *IL32*, *PDGFB*, and *IFG2BP1* between control and BETi-treated uLMS cells.

Table S1. Primers used in the study

Gene symbol	Primer sequences	F or R	Assay	Species	Product Size (bp)	Accession
CDKN1A	CGGAACAAGGAGTCAGACATT	F	q-PCR	Human	105	NM_000389.5
CDKN1A	AGTGCCAGGAAAGACAACTAC	R	q-PCR	Human	105	
CCND2	CAGGGCTGGGAGAAGAAATAG	F	q-PCR	Human	99	NM_001759.4
CCND2	CTACCTCCAATTCCCACACTAC	R	q-PCR	Human	99	
GLI1	AGCTAGAGTCCAGAGGTTCAA	F	q-PCR	Human	102	NM_005269.3
GLI1	TAGACAGAGGTTGGGAGGTAAG	R	q-PCR	Human	102	
GLI2	AGGCTGAGGTGGTCATCTAT	F	q-PCR	Human	104	DQ086814.1
GLI2	GATGTGCTCGTTGTTGATGTG	R	q-PCR	Human	104	
DNMT1	CGGCCTCATCGAGAAGAATATC	F	q-PCR	Human	95	NM_001130823.3
DNMT1	TGCCATTAACACCACCTTCA	R	q-PCR	Human	95	
DNMT3A	CTGAGGTAGCGACACAAAGTTA	F	q-PCR	Human	101	NM_175629.2
DNMT3A	CTCTTCTGGGTGCTGATACTTC	R	q-PCR	Human	101	
DNMT3B	GGAGCCACGACGTAACAAATA	F	q-PCR	Human	98	NM_006892.4
DNMT3B	GTAAACTCTAGGCATCCGTCATC	R	q-PCR	Human	98	
TET1	ACCGCTCTTTGGGTGTTATT	F	q-PCR	Human	107	NM_001406365.1
TET1	CTTGGCTTCCATTCCTTCCT	R	q-PCR	Human	107	
KDM1A	CCGATTCCACGACTCTTCTTT	F	q-PCR	Human	105	NM_001009999.3
KDM1A	TGCAATTCTTCCCGCTTCT	R	q-PCR	Human	105	
KDM1B	CTACAGATGGCACAGGGTATTC	F	q-PCR	Human	104	NM_001364614.2
KDM1B	CTTCTCTGACAACGGTGGATTA	R	q-PCR	Human	104	
SIRT1	AGAACCCATGGAGGATGAAAG	F	q-PCR	Human	111	AF083106.2
SIRT1	TCATCTCCATCAGTCCCAAATC	R	q-PCR	Human	111	
SIRT7	GCTTCCTCTGTCTGTGACTTTA	F	q-PCR	Human	93	NM_030648.4
SIRT7	AGGAGAGTTGGGAGGTATGT	R	q-PCR	Human	93	
SETD4	CTTTCTGGACCTGTGGTCTTAG	F	q-PCR	Human	109	NM_017438.5
SETD4	CACCTGTCGTTGTCAGTAGTT	R	q-PCR	Human	109	
IL32	GAGCTCTTCATGTCCTCTTTCC	F	q-PCR	Human	121	NM_001012631.4
IL32	GGCAAAGGTGGTGTCAGTAT	R	q-PCR	Human	121	
PDGFRB	CCGCATCTGTGATGAGAATGTA	F	q-PCR	Human	110	NM_002609.4
PDGFRB	GGATTCCTCCAAAGCCTCATAG	R	q-PCR	Human	110	
IGF2BP1	CCACCTGTCCCAACCATAAA	F	q-PCR	Human	103	NM_006546.4
IGF2BP1	CTAAATGGCCGACGTCTGAA	R	q-PCR	Human	103	
18S	CACGGACAGGATTGACAGATT	F	q-PCR	Human	119	NR_145820
18S	GCCAGAGTCTCGTTCGTTATC	R	q-PCR	Human	119	
